# Cotard Delusion in the Context of Schizophrenia: A Case Report and Review of the Literature

**DOI:** 10.3389/fpsyg.2016.01351

**Published:** 2016-09-07

**Authors:** Nicholas Bott, Corey Keller, Malathy Kuppuswamy, David Spelber, Joshua Zeier

**Affiliations:** ^1^Stanford University School of Medicine, Psychiatry and Behavioral SciencesStanford, CA, USA; ^2^VA Palo Alto Health Care SystemPalo Alto, CA, USA

**Keywords:** cotard delusion, schizophrenia, hyper-religiosity, violence, self-harm, traumatic brain injury (TBI)

## Abstract

**Background:** The Cotard delusion (CD) is one of a variety of narrowly defined monothematic delusions characterized by nihilistic beliefs about the body’s existence or life itself. The presence of CD within the context of schizophrenia is rare (<1%), and remains understudied.

**Case:** ‘Mr. C’ is a 58-year-old veteran with a prior diagnosis of schizophrenia, who presented with CD in the context of significant depression, suicidal ideation, violence, and self-harm behavior. He perseverated in his belief that he was physically dead and possessed by demons for several weeks. This delusion was reinforced by his religious belief that life was an attribute of God, and by inference, he as a human, was dead. His condition gradually improved over the course of treatment with Divalproex and quetiapine with discussions about the rationale for his belief. Upon discharge, Mr. C. demonstrated awareness of his fixation on death and an ability to redirect himself.

**Discussion:** This case highlights the need to better understand the co-occurrence of CD in schizophrenia, their differentiation, the increased risk of violence and self-harm behavior in this presentation, and how specific events and religious factors can influence delusional themes of CD. Pharmacotherapy and aspects of cognitive-behavioral therapy may be effective in ameliorating these symptoms in CD.

## Introduction

The Cotard delusion (CD) is one of a variety of narrowly defined monothematic delusions characterized by nihilistic beliefs about the body’s existence or life itself. It is estimated to occur in less than 1% of older adults, 3% of older adults with severe depression (Chiu, 1995), and less than 1% of patients with psychotic disorders (Ramirez-Bermudez et al., 2010; Stompe and Schanda, 2013). There are no standardized treatments for CD, although, case reports have documented effectiveness of pharmacological treatment as well as electro-convulsive treatment (Debruyne et al., 2009; Grover et al., 2014). Combination paliperidone and lorazepam has been successful in the context of schizophrenia (Morgado et al., 2015). Recent reports have also brought attention to the role cognitive-behavioral therapy (CBT) techniques for delusions may play in effective treatment (Coltheart et al., 2007). Given the unique content of beliefs in CD, additional research to understand the important role life circumstances – including religious beliefs – play in the development and maintenance of this condition is needed (Ghaffari Nejad et al., 2013).

In this report we present the case of ‘Mr. C,’ an individual with religiously mediated CD in the context of schizophrenia, we review the relevant literature on biological, psychological, and social factors associated with CD, and situate this case within all existing cases of co-occurring schizophrenia and CD.

## Case Presentation

‘Mr. C’ is a 58-year-old Navy veteran with a history of substance use disorder in sustained remission, traumatic brain injury (TBI), mild neurocognitive impairment, and a 15-year diagnosis of schizophrenia. Importantly, his sister indicated that Mr. C suffered his first psychiatric break during adolescence. He was prescribed medication, but his parents, who indicated their preference for treating his condition with prayer, refused these. He has been living with his sister for the past 20 years and participates in all activities of daily living. He is a widower and is currently unemployed. His TBI history includes a single episode approximately 25 years ago when he fell off a moving train, requiring extended hospitalization. Mr. C was brought in to our Veterans Affairs (VA) hospital by his sister after calling the Veterans’ Crisis Line with SI. One week earlier there was an altercation with his brother-in-law resulting in Mr. C attacking him with a crowbar and the sister intervening. On admission, the family reported that Mr. C was taking venlafaxine and quetiapine.

Upon initial interview, Mr. C was grossly oriented, with psychomotor agitation, mildly pressured speech, anxious mood, and restricted affect. Thought process was tangential, and his cooperation was poor due to his preoccupation with delusions that he was dead, that nothing existed. He felt his body was being energized by demons: “These demons are my energy, I am dead, how can I have any energy?” He perseverated on this content despite attempts to engage him: “How can you help me, I am dead.” When asked about suicidal ideation (SI) he indicated, “I am dead, how can I be suicidal?” When providers commented on the novelty of speaking with a dead person, he replied, “Maybe you are dead too.” Importantly, his nihilistic beliefs included strong religiously mediated content not shared by his family. Mr. C. made the comments: “That physical God is alive because he has a physical body, but the demons inside me keep me dead, keep me from being alive.” “It is not nice meeting the devil, all the demonic spirits are tormenting me and raping me.” He believed his insides were being corroded by demons and indicated that he had repeatedly sodomized himself to try and purge himself of the demons. Mr. C’s sister indicated that the onset of his religiously themed delusional symptoms began after his TBI when he became convinced that a miracle had occurred during the accident and he became aware of his “dead nature.”

**FIGURE 1:**
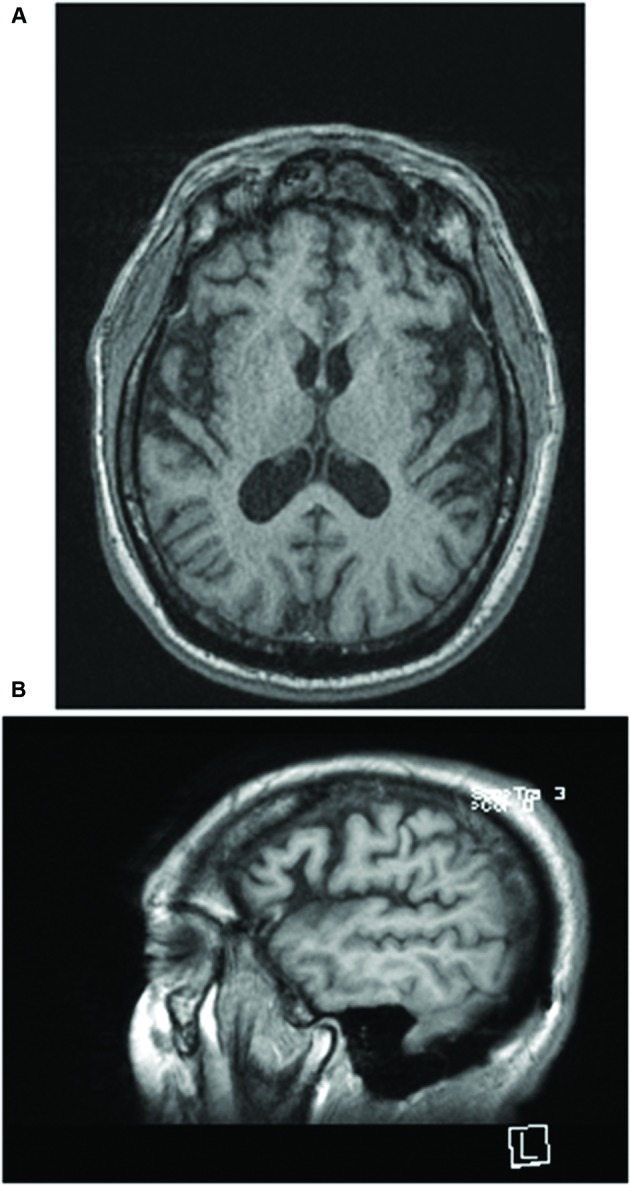
**(A)** T1 axial and **(B)** sagittal images of the brain obtained 9 months earlier demonstrate mild global volume loss for age with widening of the Sylvian fissure bilaterally. Neither evidence of past acute TBI nor white matter abnormalities were noted on fluid-attenuated inversion recovery (FLAIR) imaging.

Magnetic resonance imaging (MRI) performed in April 2015 revealed mild global volume loss for age with widening of the Sylvian fissure bilaterally. Neither evidence of past acute TBI or white matter abnormalities were noted on fluid-attenuated inversion recovery (FLAIR) imaging (**Figure 1**). Neuropsychological testing administered during the index hospitalization was significant for impairment on tasks of processing speed, simple attention, verbal learning and memory, aspects of executive functioning (set-shifting, verbal fluency), facial recognition, and affect naming and recognition. Aspects of visuospatial discrimination and construction, confrontation naming, and visual memory were intact (**Table 1**).

**Table 1:** Summary of patient performance on paper-and-pencil neuropsychological tests.

Test	Raw	%	Functional domain
WRAT-4 reading	53	14	Estimated premorbid intelligence
MoCA	15	NA	Global cognitive screen
RBANS digit span	8	15	Attention
RBANS coding	30	3	Attention, processing speed
Trails A	135”	<1	Attention, processing speed
Trails B	360” (3E)	<1	Set-shifting, processing speed
RBANS naming	10	75	Language
RBANS semantic fluency	10	1	Executive function (verbal fluency)
RBANS figure copy	19	72	Visuospatial construction
RBANS line orientation	18	71	
RBANS list learning	19	<1	Verbal learning
RBANS story learning	9	4	
RBANS list recall	2	3	Verbal memory
RBANS list recognition	19	31	
RBANS story recall	2	1	
RBANS figure recall	13	44	
BFRT	39	8	Socio-emotional
FAB affect recognition	12	<1	Socio-emotional
FAB affect naming	16	<1	Socio-emotional
IPSAQ eternalizing bias	-7	Minimal	Attributional style
IPSAQ personalizing bias	3	Severe	Attributional style
BDI-II	24	Moderate	Depression

*BDI-II, Beck Depression Inventory–II; BFRT, Benton Facial Recognition Test; FAB, Florida Assessment Battery; IPSAQ, Internal, Personal and Situational Attributions Questionnaire; MoCA, Montreal Cognitive Assessment; RBANS, Repeatable Battery for the Assessment of Neuropsychological Status; WRAT-4, Wide Range Assessment Test–4.*

Upon initial presentation, given the patient’s aggressive and activated behavior as well as sleep difficulties, venlafaxine was discontinued and quetiapine was titrated to 700 mg. Divalproex was added during the admission for mood stabilization and titrated to 750 mg. During his 2 weeks on the inpatient ward repeated inquiry about his religious experience and his relationship to God led to changes in the content of Mr. C’s delusion from literal and concrete beliefs that he was physically dead to more abstract and metaphorical descriptions of spiritual death. His belief that only God was physically alive remained, and his belief that demons possessed his body persisted. Continued probing about the patient’s existence demonstrated further amelioration of the nihilistic content. A month into his admission when asked if his daily activities (eating, drinking, showering) were evidence that he was in fact physically alive, he indicated that he was ‘probably alive.’

Mr. C continued to be alert and oriented to person, place, and time, with evidence of increased insight into his preoccupation with his belief that he was dead. Approximately 1 month into his admission he recognized when the content of his speech began to focus on death, demons, and self-negation, at which point he would stop and remark, ‘There I go again talking about all that dead stuff.’ He was ultimately discharged after 5 weeks of hospitalization. On discharge, the patient’s brother noted that he was less delusional and more linear than at baseline.

## Discussion

This case report documents the presence of religiously reinforced CD in a 58-year-old man with a longstanding history of schizophrenia and TBI. While the presence of CD in schizophrenia is rare, the co-occurrence of monothematic delusions and schizophrenia has been documented. A two-factor theory of delusional belief has been proposed to explain monothematic delusions such as Capgras and CD consisting of abnormalities that result in the generation of delusional beliefs and abnormalities that serve to maintain these beliefs despite evidence to the contrary. While the former abnormality is unique to the delusional belief in question, the latter is hypothesized to be the result of damage to the right frontal lobe impairing the belief evaluation system (Coltheart et al., 2007). In the context of schizophrenia, the etiology of monothematic delusions likely remains due to the same combination of these cognitive impairments. Content formation within CD remains unclear and it is important to gain a better understanding of the role specific life events and cultural influences play in the formation and maintenance of CD.

With the limitations of a case report, the relative rarity of CD in schizophrenia, and the limited duration during which we treated the patient, we cannot exclude more generalized bizarre delusions seen in schizophrenia as the etiology of our patient’s presentation. However, the following features of this case are consistent with previous case presentations documenting features present in CD. The content of the delusion was circumscribed and focused on his being dead (in contrast to God) with an accompanying explanation for the animation of his body (demon possession). While Mr. C’s presentation upon admission is consistent with the hypothesized “chronic” phase of CD, the resolution of his delusion follows similar phases of recovery that have previously been reported (Yamada et al., 1999; Ghaffari Nejad et al., 2013). It is also notable that our patient suffered a TBI, and that this life-threatening event was interpreted as ‘miraculous’ and was the point in time when he realized he was, in fact, dead. Previous cases of CD subsequent to TBI have suggested a causal role of events surrounding the TBI in the development of patient beliefs (Young et al., 1992; Butler, 2000; Kundlur et al., 2007). While no focal abnormalities were found on MRI, most cases of CD including structural imaging have reported no gross structural changes (Kundlur et al., 2007).

While alterations in mood, attributional style, and cognitive dysfunction are often present in psychotic spectrum disorders (Abdel-Hamid and Brune, 2008; Reichenberg, 2010), it is notable that measures across these domains of functioning in our patient were consistent with previous reports characterizing patients with CD, including moderate depressive symptoms, an internalizing attributional style, and impairment on tasks of facial recognition, affect recognition, and affect naming. Previous reports characterizing the neuropsychological profile of CD have proposed damage to affective components of the face recognition system as the cause of impairment seen on tasks of facial recognition, affect recognition, and affect naming (Young et al., 1992; Gerrans, 2000; Kundlur et al., 2007). More general right-hemisphere cognitive dysfunction has also been observed (Debruyne et al., 2009). Young and Leafhead (1996) have proposed that the co-occurrence of disruption to the affective component of the visual recognition system with an internal attributional style results in the development of CD, and one case has supported the existence of an internal attributional style (McKay and Cipolotti, 2007). CD is also typically accompanied by depressed mood and in some cases psychotic features (Berrios and Luque, 1995; Ramirez-Bermudez et al., 2010). The influence of Persian folklore in the development of CD has recently highlighted the role that cultural religious thematic content can play in the germination of patient beliefs (Ghaffari Nejad et al., 2013).

### Cotard Delusion in Schizophrenia

Diagnosis of CD can be challenging, particularly in the context of psychosis, where the presence of delusions is common. A systematic chart review of 479 primary psychiatric inpatients over the course of 2 years (Ramirez-Bermudez et al., 2010) identified three cases of CD (0.62%). Within this sample, 150 had a diagnosis of schizophrenia, but none of these patients demonstrated co-occurring CD. Similarly, in a review of the Austrian International Study of Psychotic Symptoms in Schizophrenia, Stompe and Schanda (2013) found three cases that could be diagnosed as CD (0.87%).

Given this rarity, it is perhaps not surprising that only six cases have reported on co-occurring CD and schizophrenia (**Table 2**). Accurate diagnosis can also be difficult, and this difficulty has been illustrated by Shiraishi et al. (2004), who reported on a case of schizophrenia and co-occurring CD in a 33-year-old male. In addition to the presence of delusion of persecution and delusions of control related to specific body parts, the patient developed a monothematic delusion of non-existence, and the absence of body parts. The patient also endorsed severe depressive symptoms. In this case, Shiraishi et al. (2004) categorized this disorder as schizophrenia with CD superimposed. More recently, Morgado et al. (2015) presented a case of a 42-year-old man with an existing diagnosis of schizophrenia, who presented with nihilistic delusions concerning his body, including lack of a beating heart, loss of blood and palate, and his stomach being destroyed. However, the patient did not present with depressive symptoms and there was evidence of psychomotor agitation. Morgado et al. (2015) also categorized this disorder as schizophrenia with superimposed CD.

**Table 2:** Published reports of co-occurring cotard delusion and psychotic disorders.

Reference	Age/sex	Initial presentation	Delusional content	Violence and self-harm behavior	Treatment	Course
Ko, 1989	33, Female	Behavioral alterations; paranoia; auditory hallucinations; delusions; depression	Body mutilation; abdomen opening	Self-starvation	ECT; trifluoperazine	15 months
Caliyurt et al., 2004	27, Male	Shortness of breath; heartburn; constipation; headache; insomnia	Denial of stomach; non-functional GI system and heart	Self-starvation	18 sessions ECT; olanzapine 10 mg/days	21 days
Shiraishi et al., 2004	33, Male	Delusions; depersonalization; depression	Denial of body parts; denial of bodily existence	NA	Haloperidol 10 mg/days; sulpiride 300 mg/days	5 months
Huber and Agorastos, 2012	32, Male	Agitation; delusions aggression and violent behavior	Denial of bodily existence; body reanimation by zombies	Aggression and violence toward others	Haloperidol; diazepam; clozapine	NA
Ghaffari-Nejad et al., 2007	32, Female	Self-harm; delusions; visual hallucinations	Denial of bodily existence	Cut off tip of nose	12 sessions ECT; risperidone 10 mg/days	NA
Morgado et al., 2015	42, Male	Behavioral alterations; paranoid speech; delusions; euthymic; increased psychomotor activity	“My heart does not beat,” “I have no blood,” “my palate disappeared”	NA	Paliperidone 12 mg/days and lorazepam 5 mg/days	24 days

While violent aggression and self-harm behavior can be seen in schizophrenic patients, including reports of violent crime and genital self-mutilation (Martin and Gattaz, 1991; Krasucki et al., 1995; Teixeira and Dalgalarrondo, 2009), there is evidence to suggest that co-occurring CD and schizophrenia may increase the risk of aggression and self-harm due to the specific nihilistic content of the delusions. Taking action, or refraining from taking action, as a consequence of a delusional belief has previously been shown to differentiate psychotic patients committing violent acts from those who did not (Teixeira and Dalgalarrondo, 2009). Huber and Agorastos (2012) reported a case of CD in which the patient exhibited aggression and violence toward others. The patient believed that he had drowned and because he was dead could act violently toward others without fear of retribution or legal prosecution. Nihilistic beliefs about the existence of one’s body or of life itself may lower ones inhibition for violence.

Three cases of co-occurring CD and schizophrenia with self-harm have previously been reported. Ghaffari Nejad et al. (2013) reported a case of a 32-year-old woman with co-occurring CD and schizophrenia, who cut off the tip of her nose as a form of cosmetic surgery. Caliyurt et al. (2004) reported a case of 27-year-old man with co-occurring CD and schizophrenia who engaged in self-starvation as a result of his belief that he had no stomach and that his heart was no longer beating. Ko (1989) also reported a case of a 33-year-old woman, who engaged in self-starvation as a result of her belief that her intestines had been mutilated and that her abdomen was opening up. The present case is consistent with these reports. Mr. C responded violently to his delusion that demons were “corroding” his body through multiple incidents of self-sodomy. Furthermore, Mr. C demonstrated violent behavior toward his brother-in-law, which precipitated his hospitalization.

## Conclusion

Our case of Mr. C underscores the roles of specific life-threatening events (TBI) and religious beliefs in the content formation of CD. It also adds to previous reports documenting the increased risk of self-harm associated with co-occurring schizophrenia and CD. Given this increased susceptibility for violent acts and self-harm, the presence of delusions characterized by nihilistic beliefs about the body’s existence or life itself should prompt vigilance and clinical assessment of violent or self-harm behavior. This case also provides anecdotal evidence for the efficacy of a combination of pharmacotherapy and CBT for the treatment of monothematic delusional beliefs, including those seen in the context of psychosis. While co-occurring CD and schizophrenia is rare, the consistency and longevity of the delusional content, increased risk of self-harm behavior, presence of significant depressive symptoms, attributional style, and neuropsychological performance can each help to identify CD within this population.

## Ethics Statement

Written informed consent was obtained from the patient for publication of this case report and any accompanying images.

## Author Contributions

NB made a substantial contribution to the design of the work, the acquisition and interpretation of the work, drafted the work, approved the final version to be published and agreed to be accountable for all aspects of the work. CK made a substantial contribution to the acquisition and interpretation of the work, revised the work critically for important intellectual content, approved the final version to be published and agreed to be accountable for all aspects of the work. MK made a substantial contribution to the acquisition and interpretation of the work, revised the work for important intellectual content, approved the final version to be published and agreed to be accountable for all aspects of the work. DS made a substantial contribution to the acquisition and interpretation of the work, approved the final version to be published and agreed to be accountable for all aspects of the work. JZ made a substantial contribution to the acquisition and interpretation of the work, revised the work for important intellectual content, approved the final version to be published and agreed to be accountable for all aspects of the work.

## Conflict of Interest Statement

The authors declare that the research was conducted in the absence of any commercial or financial relationships that could be construed as a potential conflict of interest.
